# Transcriptomic Profiling of High-Density *Giardia* Foci Encysting in the Murine Proximal Intestine

**DOI:** 10.3389/fcimb.2017.00227

**Published:** 2017-05-31

**Authors:** Jonathan K. Pham, Christopher Nosala, Erica Y. Scott, Kristofer F. Nguyen, Kari D. Hagen, Hannah N. Starcevich, Scott C. Dawson

**Affiliations:** ^1^Department of Microbiology and Molecular Genetics, University of California, DavisDavis, CA, United States; ^2^Department of Animal Science, University of California, DavisDavis, CA, United States

**Keywords:** *in vivo Giardia*, transcriptome, mouse model, encystation, oxidative stress

## Abstract

*Giardia* is a highly prevalent, understudied protistan parasite causing significant diarrheal disease worldwide. Its life cycle consists of two stages: infectious cysts ingested from contaminated food or water sources, and motile trophozoites that colonize and attach to the gut epithelium, later encysting to form new cysts that are excreted into the environment. Current understanding of parasite physiology in the host is largely inferred from transcriptomic studies using *Giardia* grown axenically or in co-culture with mammalian cell lines. The dearth of information about the diversity of host-parasite interactions occurring within distinct regions of the gastrointestinal tract has been exacerbated by a lack of methods to directly and non-invasively interrogate disease progression and parasite physiology in live animal hosts. By visualizing *Giardia* infections in the mouse gastrointestinal tract using bioluminescent imaging (BLI) of tagged parasites, we recently showed that parasites colonize the gut in high-density foci. Encystation is initiated in these foci throughout the entire course of infection, yet how the physiology of parasites within high-density foci in the host gut differs from that of cells in laboratory culture is unclear. Here we use BLI to precisely select parasite samples from high-density foci in the proximal intestine to interrogate *in vivo Giardia* gene expression in the host. Relative to axenic culture, we noted significantly higher expression (>10-fold) of oxidative stress, membrane transporter, and metabolic and structural genes associated with encystation in the high-density foci. These differences in gene expression within parasite foci in the host may reflect physiological changes associated with high-density growth in localized regions of the gut. We also identified and verified six novel cyst-specific proteins, including new components of the cyst wall that were highly expressed in these foci. Our *in vivo* transcriptome data support an emerging view that parasites encyst early in localized regions in the gut, possibly as a consequence of nutrient limitation, and also impact local metabolism and physiology.

## Introduction

*Giardia lamblia* is a zoonotic protozoan parasite that causes acute and chronic diarrheal disease, primarily in areas lacking adequate sanitation and water treatment (Savioli et al., 2006; Troeger et al., 2007), and commonly affects travelers and immunosuppressed individuals (Adam, 2001). Giardiasis is a serious disease of children, who may experience substantial morbidity including diarrhea, malnutrition, wasting, and developmental delay (Solaymani-Mohammadi and Singer, 2010; Bartelt et al., 2013; DuPont, 2013). New therapeutic treatments for this widespread and neglected diarrheal disease are needed as there are estimated failure rates of up to 20% for standard treatments (Upcroft and Upcroft, 2001) and reports of drug resistance (Upcroft et al., 1996; Barat and Bloland, 1997; Land and Johnson, 1999).

Giardiasis may be either acute and/or chronic, and infection is generally accompanied by abdominal cramps, gas, nausea, and weight loss. Giardiasis may also result in a severe form of malabsorptive diarrhea presenting as a fatty, watery stool (Nosala and Dawson, 2015). Trophozoites are not invasive and giardiasis does not produce a florid inflammatory response; however, giardiasis is associated with villus shortening, enterocyte apoptosis, hypermobility, and intestinal barrier dysfunction (Halliez and Buret, 2013). The mechanisms by which *Giardia* colonization of the gastrointestinal tract induces diarrheal disease remain unclear.

*Giardia* differentiates into two morphological forms during its life cycle: a motile, multi-flagellated trophozoite that colonizes the host small intestine, and an infective cyst that is shed into the environment (Heyworth, 2014). The *Giardia* cyst is characterized by four nuclei and a thick outer cell wall consisting of cyst wall proteins 1, 2, and 3 and a unique β-1,3- linked N-acetylgalactosamine homopolymer. Other components of the cyst include protein disulfide isomerases (Davids et al., 2004), high cysteine membrane proteins (HCMPs), and EGFP family proteins (Davids et al., 2006). The structure of the cyst wall allows cysts to persist in the environment until ingested by a compatible host (Gillin et al., 1989; Lujan et al., 1996, 1997; Lauwaet et al., 2007; Faso and Hehl, 2011). Following ingestion, cysts transform into motile trophozoites as they pass into the gastrointestinal tract. Trophozoites navigate the lumen of the small intestine and attach to the microvilli of the small intestine via the ventral disc, yet do not invade the epithelium (Adam, 2001). Attachment to the gut epithelium allows *Giardia* to resist peristaltic flow and proliferate in this low oxygen, nutrient rich environment. Encystation can be induced *in vitro* by increasing pH and decreasing cholesterol, or by increasing bile and lactic acid in the medium (Gillin et al., 1988, 1989; Lujan et al., 1997). However, cysts produced by *in vitro* methods tend to excyst less efficiently (Boucher and Gillin, 1990) and are less robust at establishing infections in animal models than cysts harvested directly from feces. Determining the additional factors required for robust differentiation to *in vivo* cysts is critical toward understanding *in vivo* host-parasite interactions associated with giardiasis (Morf et al., 2010; Faso et al., 2013; Sulemana et al., 2014).

The lack of accessibility to the gastrointestinal tract (Lujan et al., 1996, 1997, 1998) has limited our understanding of *in situ Giardia* physiology in the host. Parasite physiology and metabolism have been inferred from axenic laboratory culture in a non-defined medium (Keister, 1983) or from co-culture with intestinal epithelial cell lines (Inge et al., 1988; Lujan et al., 1996, 1998). However, co-incubation studies of trophozoites with intestinal cell lines may not accurately reflect *in vivo* parasite physiology. *Giardia* infects many mammalian hosts, and animal models of giardiasis include adult (Byrd et al., 1994; Singer, 2016) or suckling mice (Mayrhofer et al., 1992) or adult gerbils (Rivero et al., 2010) infected with either *Giardia lamblia or G. muris* isolates (Aggarwal and Nash, 1987). While the adult mouse model of giardiasis is commonly used to evaluate anti-giardial drugs (Miyamoto and Eckmann, 2015), previous *in vivo* parasite studies lack precision due to tissue sampling throughout the intestinal tracts of infected animals without corresponding knowledge of *Giardia* colonization.

Our recent development of methods for non-invasive imaging of bioluminescent *Giardia* parasites permits unprecedented access to real-time host-parasite interactions, allowing us to reexamine decades-old assumptions about *Giardia* infection and encystation dynamics in living hosts (Barash et al., 2017b). BLI enables sensitive quantification and live reporting of transcriptional activity with promoter-luciferase fusions (Weissleder and Ntziachristos, 2003; Welsh and Kay, 2005; Luo et al., 2006) and protein abundance and stability with protein-luciferase fusions (Stacer et al., 2013). BLI is used commonly to monitor parasitic infection dynamics during malaria, leishmaniasis, trypanosomiasis, and toxoplasmosis (Saeij et al., 2005; D'Archivio et al., 2013; Reimao et al., 2013), as well as bacterial colonization of the intestine (Hutchens and Luker, 2007). The use of imaging methods to evaluate *in vivo* giardiasis has allowed the precise longitudinal and spatial monitoring of the dynamics of infection, and provides an improved method to evaluate anti-giardial drugs in a relevant animal model of giardiasis.

Recently we used non-invasive imaging of bioluminescent *Giardia* parasites to evaluate real-time parasite physiology in the host, and confirmed early observations of *G. muris* and *G. lamblia* colonization of the proximal small intestine of mice (Owen et al., 1979), gerbils (Campbell and Faubert, 1994), and humans (Oberhuber et al., 1997). By imaging mice infected with *Giardia* expressing firefly luciferase under the control of encystation-specific promoters (Wiles et al., 2006; Hutchens and Luker, 2007; Andreu et al., 2011; Gourguechon and Cande, 2011; Rhee et al., 2011) we determined that encystation is initiated early in the course of infection and is correlated to high-density regions, or foci. These foci persist in the gastrointestinal tract throughout the course of infection.

Here we interrogate the *in vivo* physiology of high-density *Giardia* foci in the proximal small intestine and compare this to physiology in axenic culture. We also demonstrate that parasite metabolism in the foci is defined by the upregulation of encystation genes and of genes involved in oxidative stress responses. Furthermore, we observe that the expression profiles of encysting trophozoites in high-density foci are similar to profiles seen during mid-to-late encystation (7–22 h in *in vitro* encystation medium) (Einarsson et al., 2016). Finally, we identify and confirm six new encystation genes that are highly expressed in the foci.

## Materials and methods

### Giardia trophozoite and encystation culture conditions

The *G. lamblia* (ATCC 50803) bioluminescent reporter *P*_GDH_-*Fluc* strain (Barash et al., 2017b) all C-terminal GFP-fusion strains, and the wild type WBC6 strain were cultured in modified TYDK medium (also known as TYI-S-33 or Keister's medium; Keister, 1983) supplemented with bovine bile and 5% adult and 5% fetal bovine serum (Trapnell et al., 2010) in sterile 16 ml screw-capped disposable tubes (BD Falcon), and incubated upright at 37°C without shaking. Encystation was induced *in vitro* by decanting TYDK medium from 24-h cultures (roughly 30% confluent) and replacing with encystation medium modified by the addition of 0.5 grams/liter bovine bile, pH 7.8 (Carpenter et al., 2012). After 24 h, cysts settled and were harvested for subsequent imaging analyses.

### *In vivo* and *Ex vivo* bioluminescence imaging of mice with spatial sampling of *Giardia* colonization

Animal studies were performed with IACUC approval at the University of California, Davis (Scott C. Dawson, PI). Protocols for *in vivo and ex vivo* bioluminescence imaging of *Giardia*-infected mice were as previously described (Barash et al., 2017b). Specifically, four 8 week old, female C57BL/6J mice (Jackson Laboratories) were maintained on *ad libitum* water and alfalfa-free irradiated rodent pellets (Teklad 2918). Water was supplemented with 1 mg/ml ampicillin and neomycin (Teknova) for 5 days prior to infection (Solaymani-Mohammadi and Singer, 2011) to promote parasite colonization, and mice were maintained on antibiotics for the entire experiment. For infections, each animal was gavaged with 1 × 10^7^ *G*. *lamblia P*_*GDH*_-*Fluc* strain trophozoites in 100 μL phosphate-buffered saline (Barash et al., 2017b).

For non-invasive monitoring of parasite colonization using *in vivo* BLI, mice were sedated, D-luciferin (30 mg/kg) was then injected intraperitoneally, and bioluminescence was imaged using an IVIS Spectrum (PerkinElmer) with an open emission filter (Barash et al., 2017b). Photons were quantified using an ultra-sensitive CCD camera (IVIS Spectrum) and the resulting heat maps of bioluminescent photon emission intensity were overlaid on still images of anesthetized animals. To allow the D-luciferin to distribute throughout the body, images were collected with 2-min exposures constantly over 8–10 min until the bioluminescent signal stabilized. The exposure time for final image collection ranged from 2 to 5 min due to differences in the signal strength. Bioluminescence was quantified using LivingImage software to draw a region of interest (ROI) around each mouse, from front paws to anus. BLI data was quantified as total flux (photons/second) for exposure time-independent quantification of signal intensity.

To image the *ex vivo* spatial location and density of *Giardia* in the murine gastrointestinal tract, sedated mice were euthanized on days 3 or 7 post-gavage by cervical dislocation. The entire gastrointestinal tract was quickly dissected from esophagus to anus and positioned within a plastic Petri dish for *ex vivo* imaging using thirty second exposures (Barash et al., 2017b). ROI analysis was used to quantify bioluminescence (LivingImage). Total gastrointestinal tract signal was analyzed within a circle encompassing the entirety of the petri dish. The stomach, proximal SI (first half), distal SI (second half), cecum, and large intestine were traced using the free-hand tool and total flux was recorded as stated above (Supplemental Table 1). Spatial imaging of bioluminescence in the entire gastrointestinal tract specified the particular intestinal segments enriched for high-density *Giardia* foci of colonization. Small intestinal segments with such foci were excised for downstream RNA extraction and stored in RNAlater (Life Technologies) at −80°C, or alternatively, were fixed in 10% phosphate-buffered formalin for histology.

Histological sections of these intestinal samples were stained and visualized using light microscopy, confirming the presence of *Giardia* trophozoites in selected intestinal samples (Barash et al., 2017b). Protocols for preparation of histological slides were the same as those described in Chen et al. (2015) and were performed by the UC Davis Anatomic Pathology and IHC Laboratory (Davis, CA). Briefly, a subset of the excised bioluminescent intestinal samples was fixed in 10% phosphate-buffered formalin for 24 h. Specimens were dehydrated in a series of graded ethanol (70, 96, and 99%) and embedded in paraffin. Five-micron sections were cut perpendicular to the mucosa surface and the paraffin was cleared from the slides with coconut oil (over 15 min, 60°C). Sections were rehydrated in 99, 96, and 70% ethanol followed by a 10 min wash in water and stained with hematoxylin and eosin (HE). All HE slides were visualized via brightfield microscopy using a Leica DMI 6000 wide-field inverted fluorescence microscope with PlanApo 10X and 40X objectives.

### Total RNA extraction and RNA-Seq of gastrointestinal samples in infected animals

Total RNA was extracted by from *Giardia*-infected intestinal segments by resuspending the tissue in RNA STAT 60 solution (Tel-Test, Inc.) and repeatedly pipetting through a glass pipette until tissue homogenization was complete. All samples were kept on ice during tissue homogenization. Total RNA was purified by phenol-chloroform extraction and isopropanol precipitation. Poly(A) selection was performed for both the *in vivo* and *in vitro* samples with one microgram of total RNA. Illumina sequencing libraries were constructed and ribosomal RNA was depleted (UC Davis DNA Technologies Core Facility, Davis, CA) for downstream Illumina sequencing. 120–180 million 50 bp, single end reads were subsequently generated via Illumina sequencing (HiSeq 2500 system). All *Giardia* reads were submitted to the NCBI Sequence Read Archive (ID: SRP104997), with SRA accession numbers SRX2754297 to SRX2754300 for *in vivo* samples and SRX2754301 to SRX2754304 for *in vitro* samples.

### Trimming and processing of transcriptome sequences

**Figure 1:**
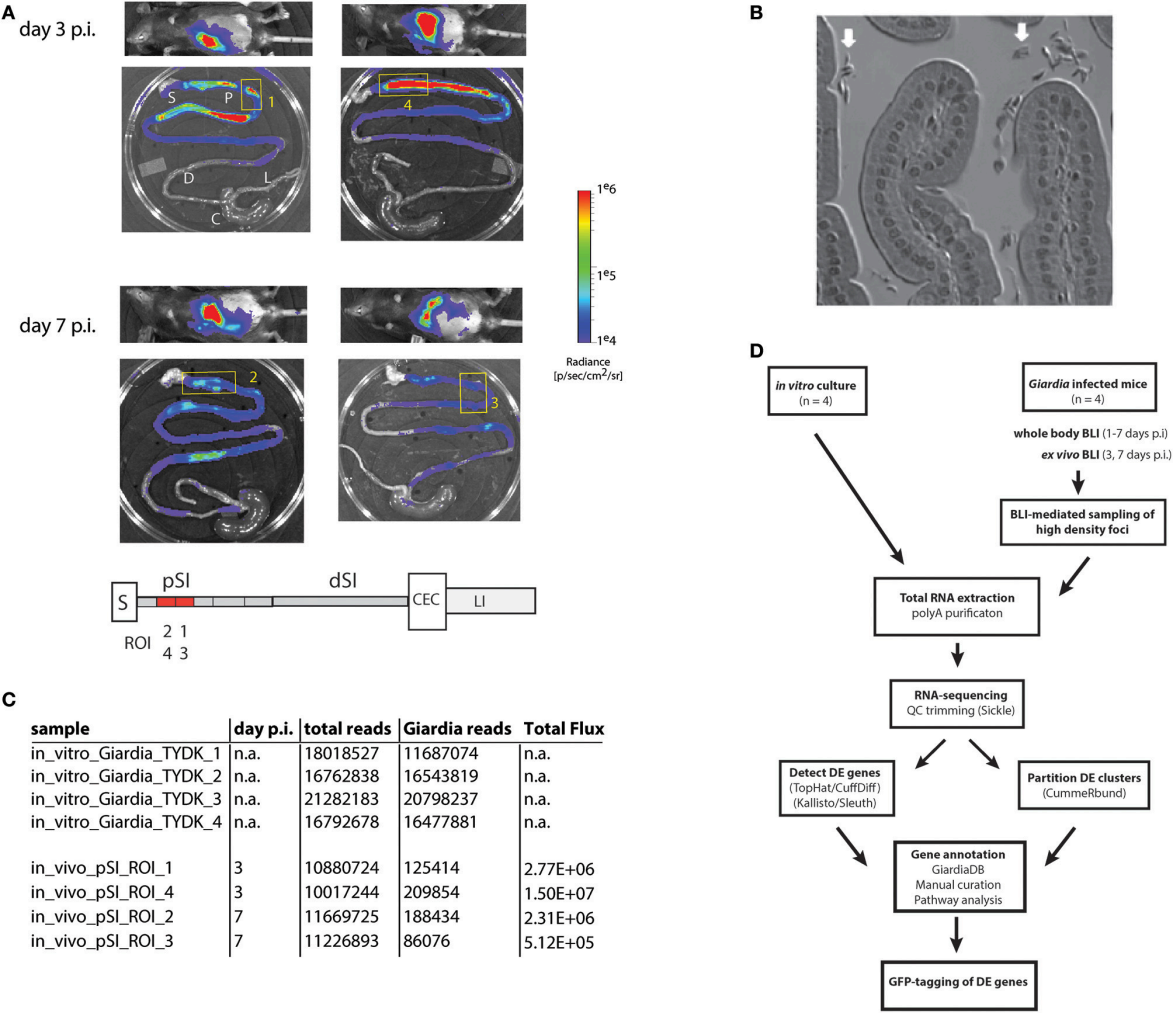
Bioluminescent imaging allows precise sampling of *Giardia* foci in the proximal small intestine for transcriptomic analyses. A cohort of four mice were infected with one million trophozoites by gavage of the *P*_*GDH*_-*FLuc Giardia* strain (Barash et al., 2017b). Whole body images were non-invasively collected for parasite bioluminescence from the constitutive *P*_*GDH*_-*FLuc* bioreporter **(A)**, and mice were subsequently sacrificed at 3 or 7 days post-infection (p.i.). Image overlays display the *P*_*GDH*_-*FLuc* bioluminescence intensity, with the highest signal intensity shown in red and the lowest in blue. The colored radiance scale indicates the photon flux (p/s/cm^2^/sr) for each intestinal segment. *Ex vivo* imaging defined four foci in the proximal small intestine (yellow numbered boxed regions) subsequently excised for transcriptomic sequencing and analysis **(A)**. In the schematic, regions of the gastrointestinal tract are marked as: S, stomach; pSI, proximal small intestine; dSI, distal small intestine; CEC, cecum; and LI, large intestine, with the positions of the *Giardia* foci (ROI1-ROI4) within the excised fragments in red. In **(B)**, the enrichment of *Giardia* trophozoites (white arrows) in a representative bioluminescent small intestinal sample was verified using light microscopy of histological sections. **(C)** Summarizes the total transcriptomic reads for each *in vitro* culture replicate (TYDK) and for each transcriptomic sample (ROI1-ROI4) from the proximal small intestine (pSI). The total reads include those from the host (mouse) before the *Giardia* reads were computationally mapped to the *Giardia* genome. The total flux (p/s/cm^2^/sr) was quantified for that particular sample. The overall transcriptomic analysis is summarized in **(D)** (DE = differentially expressed).

Raw sequences were trimmed using the sliding window trimmer Sickle (Joshi and Fass, 2011). Two pipelines were used for mapping and transcript quantification (Figure 1D). First, the reads were mapped to the *Giardia* assemblage A genome (WBC6, release 5.0) using TopHat (version 2.0.14) (Trapnell et al., 2009). The TopHat output BAM files were processed and assembled into transcripts by Cufflinks (version 2.2.1) (Trapnell et al., 2010) and normalized to fragments per kilobase of transcript per million fragments mapped (FPKM, see Supplemental Table 2). Differentially expressed genes were identified using the Cuffdiff function of the Cufflinks package (version 2.2.1) (Trapnell et al., 2012). Differentially expressed genes were defined using a threshold cutoff false-discovery rate (FDR) of 5%. The R package cummeRbund was used to generate summary figures for the resulting RNA-seq data, such as heat maps, PCA plots, and gene clusters of differentially expressed genes (via partitioning tools) (Trapnell et al., 2012). The second pipeline mapped the reads back to the *Giardia* transcriptome (GiardiaDB-5.0_GintestinalisAssemblageA.gff) with Kallisto (Bray et al., 2016), using the recommended 30 bootstraps per sample. The companion package, Sleuth (Bray et al., 2016), was used for differential gene expression analysis, using the Wald test and significance *q*-value cutoff of 0.05. Transcripts showing concordant significant differential gene expression between Cuffdiff and Sleuth statistical packages were selected as confident differentially expressed (DE) genes.

For visualization, FPKM values of the 475 confident DE genes were hierarchically clustered. Bi-clustering was performed by Pearson correlation for transcript expression and Spearman correlation of expression profiles associated with each sample. The heatmap (Supplemental Table 3) was plotted using the heatmap.2 function of “gplots” the R software package (https://CRAN.R-project.org/package=gplots).

### Functional transcriptome annotation, metabolic pathway mapping, and partitioning analyses

Cellular functions were inferred for significant differentially expressed *Giardia* genes using analytical tools associated with the GiardiaDB (http://giardiadb.org/giardiadb/; Aurrecoechea et al., 2009), with subsequent manual inspection and curation of homology, functional domains, and cellular process designations according to prior experimental and bioinformatic evidence. GiardiaDB analytical tools were used to compare *in vivo* transcriptomic reads to prior transcriptomic and proteomic experimental studies (Palm et al., 2005; Morf et al., 2010; Ringqvist et al., 2011). In addition, the cummeRbund R package was used for partitioning analysis to predict nine differentially expressed gene clusters using the Jenson-Shannon distance binning methods with the cummeRbund software package (Trapnell et al., 2012), and compared to both the *in vivo* foci gene clusters and prior *Giardia* encystation and Caco-2-associated transcriptome studies (Palm et al., 2005; Morf et al., 2010; Ringqvist et al., 2011).

### C-terminal GFP fusion strain construction of select differentially expressed genes

To confirm the cellular localization of novel differentially expressed genes identified in the *in vivo* transcriptome, 15 differentially expressed *Giardia* genes (GL50803_7797, GL50803_4705, GL50803_5258, GL50803_7710, GL50803_15499, GL50803_10822, GL50803_15427, GL50803_24412, GL50803_14748, GL50803_6679, GL50803_88960, GL50803_5515, GL50803_14567, GL50803_14926, GL50803_9354) were GFP-tagged via our laboratory's Gateway cloning pipeline (Dawson and House, 2010). We also tagged 14 genes that were more highly expressed in *in vitro* culture. The C-terminal GFP fusion constructs included approximately 200–250 nucleotides upstream of the gene, the gene itself in frame with GFP, and a puromycin resistance cassette (Dawson and House, 2010). The *Giardia* strain WBC6 was electroporated with 20 μg of GFP-fusion plasmids, and transformed strains were maintained under antibiotic selection (50 μg/ml puromycin) for at least 2 weeks (Dawson and House, 2010).

### Immunostaining and imaging of encysting GFP-tagged strains

To visualize encystation vesicles and the mature cyst, GFP-tagged trophozoites were encysted using established *in vitro* encystation methods (Carpenter et al., 2012). Sterile deionized water was then added to all experimental culture tubes to lyse incompletely encysted cells. Water resistant cysts were stored at 4°C for subsequent imaging. Cyst wall protein 1 (CWP1) was visualized by immunostaining fixed encysting trophozoites attached to coverslips using a 1:200 dilution of anti-CWP1 primary antibody (Waterborne, Inc., New Orleans, LA) and a goat anti-mouse antibody conjugated to Alexa Fluor 594 (Invitrogen) as previously described (Carpenter et al., 2012). Coverslips were mounted onto slides using Prolong Gold Antifade Solution with DAPI (Invitrogen).

Three dimensional stacks of immunostained samples were acquired using the Metamorph image acquisition software (MDS Technologies) with a Leica DMI 6000 wide-field inverted fluorescence microscope with a PlanApo 100X, NA 1.40 oil immersion objective. Serial sections of GFP-tagged strains were acquired at 0.2 μm intervals and deconvolved using Huygens Professional deconvolution software (SVI). Two dimensional maximum intensity projections were created from the 3D stacks for presentation.

## Results

### Bioluminescent imaging allows precise sampling of regions of the proximal small intestine with high-density *Giardia* foci for transcriptomics

A cohort of four mice was inoculated with the constitutive metabolic bioreporter strain *P*_*GDH*_-*FLuc* (Barash et al., 2017b). *Giardia*-specific bioluminescence was quantified non-invasively in animals from 1 to 7 days post-inoculation (Figure 1A and Materials and Methods). Two animals were sacrificed at day 3 p.i., and two more at day 7 p.i. *Ex vivo* imaging of the gastrointestinal tracts revealed dense foci of bioluminescent signal primarily in the proximal and distal small intestine (Figure 1A); bioluminescent flux directly correlates with the number of active *Giardia* parasites (Barash et al., 2017b). Proximal intestinal segments from each gastrointestinal tract were sampled, and the bioluminescent flux of the *Giardia* foci (5.12 × 10^5^ to 1.5 × 10^7^ photons/second/cm^2^; Figures 1A,C) was determined. Representative proximal small intestinal samples (ROI) from each gastrointestinal tract were selected for transcriptomic analyses. The histology of these segments indicated no verifiable host tissue damage (Materials and Methods), but did indicate obvious colonization of these regions by *Giardia* trophozoites (Figure 1B).

PolyA-purified total RNA was extracted from four *in vivo* intestinal samples and was used to create RNA-seq libraries for sequencing (Materials and Methods and Figures 1C,D). Four additional RNA-seq libraries were created and sequenced from four independent *in vitro Giardia P*_*GDH*_-*FLuc* cultures grown axenically in standard medium (Materials and Methods). Raw sequence reads generated for the RNA-seq samples ranged from 10.0 to 21.3 million (Figure 1C). The majority of RNA-seq reads from the *in vitro* culture (TYDK) datasets (97.7–98.7%) were mapped back to the WBC6 genome. Due to the high proportion of host total RNA in the *ex vivo* samples, fewer RNA-seq reads (0.8–1.9%) were mapped to the WBC6 genome; however, these numbers were comparable between individual *in vivo* samples and were sufficient for the two statistical analysis pipelines to determine differential gene expression (Figure 1C and Materials and Methods).

### Identification of 475 differentially expressed (DE) genes between *In vitro* culture and *In vivo* foci transcriptomes

**Figure 2:**
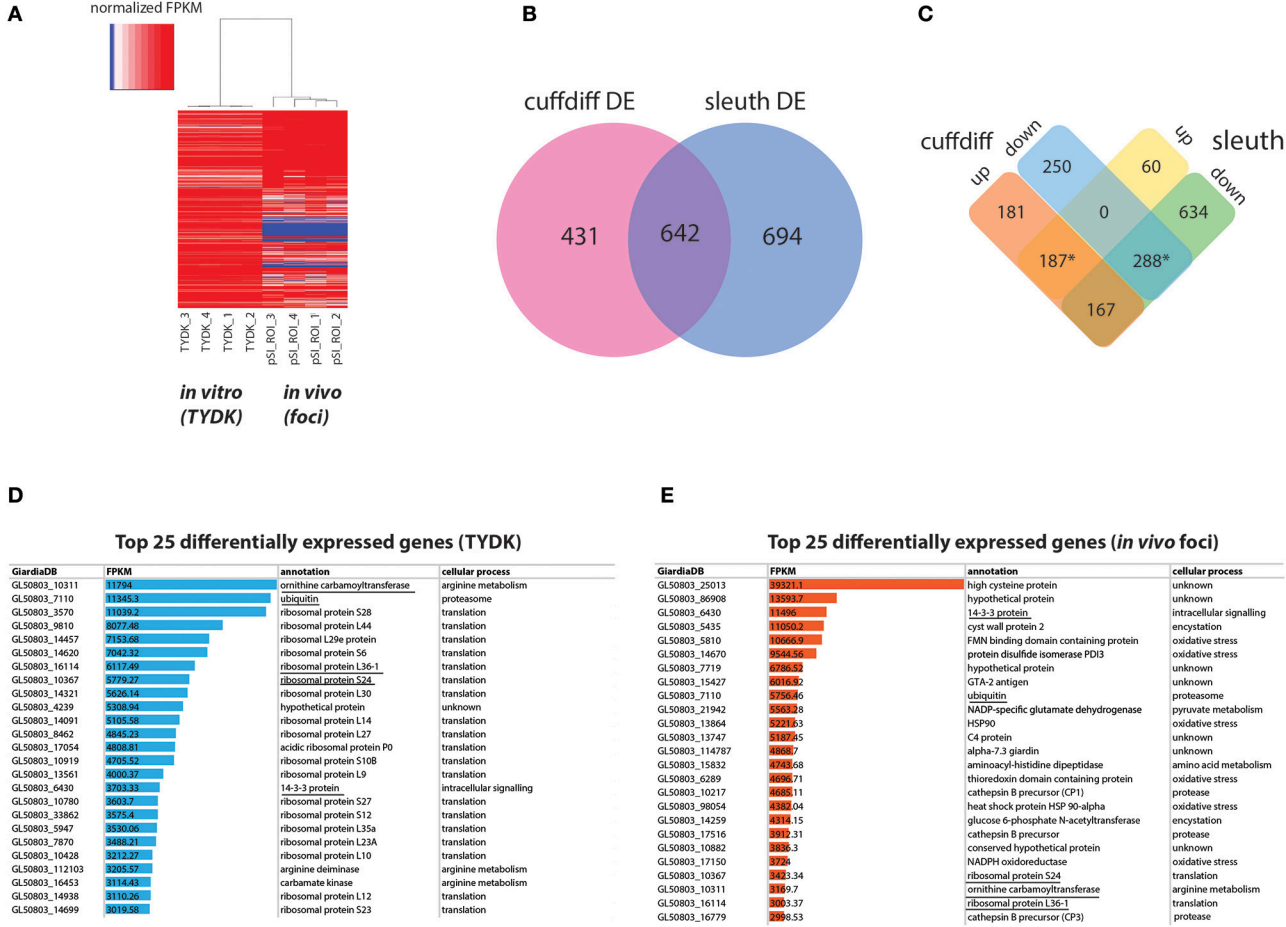
Summary of differentially expressed *in vitro* and *in vivo* foci transcriptomes using Cuffdiff and Sleuth. **(A)** Depicts a heat map of normalized FPKM comparing RNA-seq datasets from the *Giardia* grown in *in vitro* culture (TYDK) to *Giardia* transcriptomes associated with the *in vivo* foci excised from the proximal small intestine (pSI) (see Figure 1). For clarity, only differentially expressed genes confirmed by two independent methods are summarized by the heat map, with red indicating highly expressed genes, and blue indicating low expressed genes. Differentially expressed transcripts in the *in vitro* (TYDK) and *in vivo* (foci) datasets were identified by the concordance of two computational methods **(B)**. Overlap in both upregulated and downregulated genes in the *in vivo* transcriptomes was calculated and is shown in **(C)**. The asterisk indicates the 475 differentially upregulated genes (187 total) and downregulated genes (288 total) that were concordant between both methods. Of the 475 concordant differentially expressed genes, the 25 most highly transcribed genes present in the *in vitro* **(D)** and *in vivo* foci transcriptomes **(E)** are also ranked (FPKM). Underlined genes are those that are among the 25 most highly transcribed genes in both the *in vitro* and the *in vivo* transcriptome datasets.

Transcriptomic comparisons and analyses between *in vitro* and *in vivo* samples were performed using the TopHat, Cufflinks, cummeRbund, Kallisto, and Sleuth packages as outlined in the bioinformatic strategy in Figure 1D and (Trapnell et al., 2012). A comparison of the normalized read (FPKM) profiles of the *in vitro* and *in vivo* transcriptomes indicate that the four *in vitro* profiles were very similar to one another, as were the four *in vivo* foci profiles (see heat map, Figure 2A and Supplemental Table 2). Significant differences in gene expression profiles between the *in vitro* and *in vivo* samples (Figure 2A) were quantified and summarized using two different computational methods (Cuffdiff and Sleuth). Cuffdiff and Sleuth identified 1073 and 1336 differentially expressed genes, respectively, between the *in vitro* and *in vivo* datasets (Figure 2B). Four hundred seventy-five differentially expressed genes were concordant between the two methods (marked by asterisks, Figure 2C). One hundred eighty-seven of the concordant differentially expressed genes had increased expression in the *in vivo* transcriptomes relative to the *in vitro* transcriptomes (Figure 2C). Two hundred eighty-eight of the concordant differentially expressed genes had decreased expression in the *in vivo* foci transcriptomes relative to *in vitro* transcriptomes (Figure 2C). All subsequent analyses focused on the concordant genes with expression levels that varied more than 2-fold between the *in vivo* and *in vitro* transcriptomes. These included 187 genes that were more highly expressed *in vivo* foci and 166 that were more highly expressed in *in vitro* culture. (Figure 2C and Supplemental Tables 4,5). Sixty-one genes that were expressed *in vitro* were not detected in the *in vivo* foci transcriptomes (Supplemental Table 6).

Statistically significant increases in gene expression ranging from 2.5- to 153-fold were observed for *Giardia* in *in vivo* foci relative to trophozoites cultured *in vitro*. With respect to the most highly transcribed genes, transcripts associated with translation and arginine metabolism were the most transcribed categories of genes in the *in vitro* transcriptomes (Figure 2D and Supplemental Tables 4,6). In contrast, transcripts associated with encystation, oxidative stress, or unknown functions are among the most transcribed categories of genes in the *in vivo* foci transcriptomes (Figure 2E). Despite significant differences between *in vivo* and *in vitro* expression profiles, five genes (ornithine carbamoyl transferase (OCT, 10311), ubiquitin (7110), 14-3-3 protein (6430), ribosomal protein L36-1 (16114) and ribosomal protein S24 (10367) are among the top transcribed genes in both transcriptome datasets (Figures 2D,2E).

### Encystation-specific and oxidative stress genes are significantly upregulated in the *In vivo* foci

**Figure 3:**
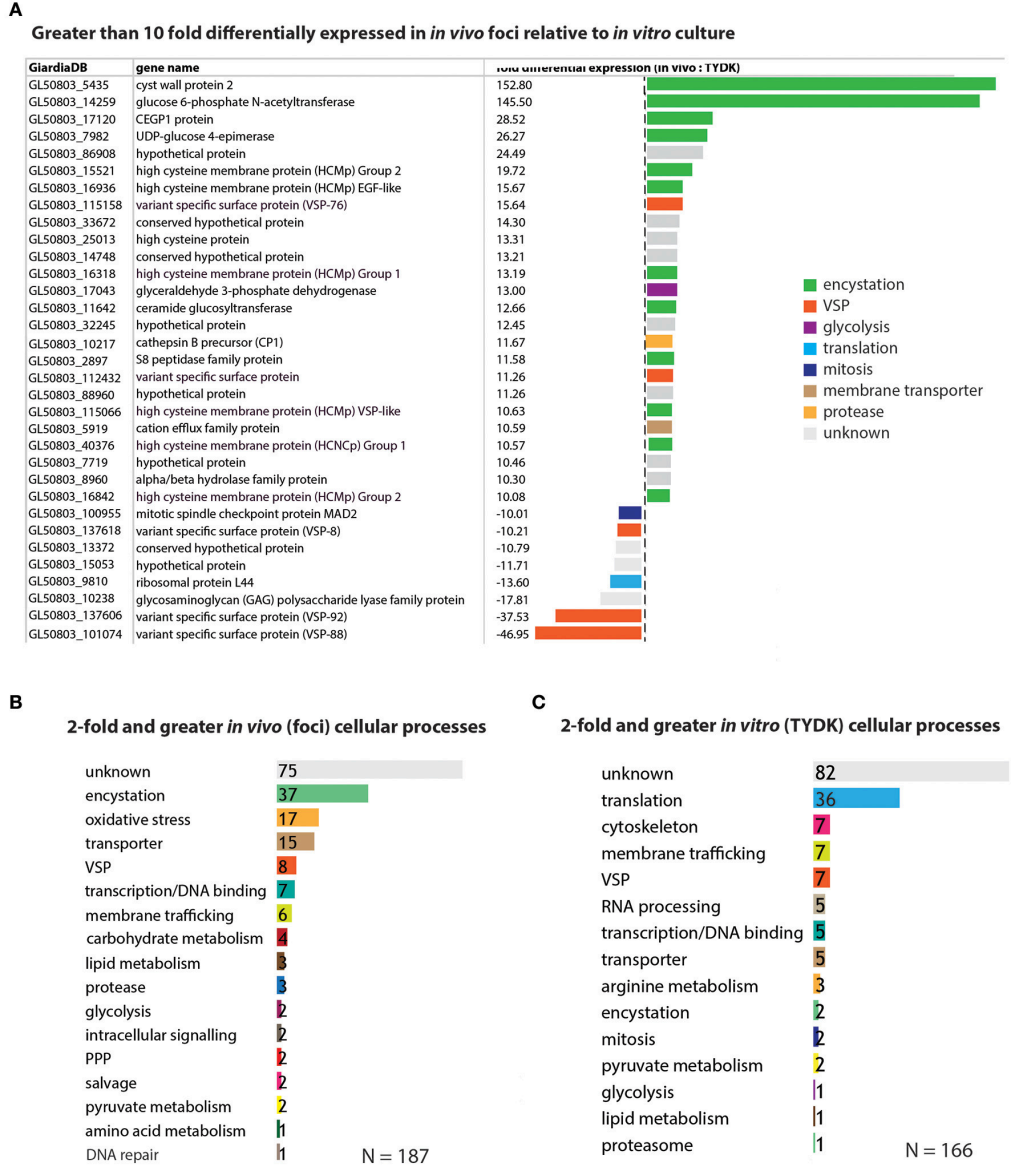
Encystation and oxidative stress genes are the top differentially expressed genes associated with the *in vivo* foci transcriptomes. In **(A)**, genes that are greater than 10-fold differentially expressed in the *in vivo* foci transcriptomes relative to *in vitro* culture are ranked and colored according to cellular process (e.g., encystation). Genes differentially expressed greater than 2-fold are ranked according to the number that are associated with a particular cellular process in the *in vivo* foci transcriptome **(B)** and the *in vitro* (TYDK) transcriptome **(C)**.

Of the 475 differentially expressed genes, 25 genes were expressed more than 10-fold higher in the *in vivo* foci relative to cultured cells; however, only 8 genes were expressed at levels more than 10-fold higher in cultured cells than *in vivo* (Figure 3A and Supplemental Tables 4,5). The expression of 85 genes was elevated more than 5-fold in foci relative to cultured trophozoites, and there were 29 genes whose expression was more than 5-fold higher in the *in vitro* transcriptomes relative to the *in vivo* transcriptomes (Supplemental Tables 4,5).

In contrast to the genomes of model organisms, many genes within the WBC6 genome lack functional gene annotations associated with *Giardia*-specific processes (e.g., encystation or pyruvate metabolism). Thus, we manually curated the functional annotations of genes whose expression was more than 2-fold higher *in vivo* (187 genes) or *in vitro* (166 genes) and summarized the functional categories associated with these statistically significant differentially expressed genes (Figure 3). Ten of the 25 genes with more than 10-fold higher expression in the *in vivo* foci are associated with encystation-specific processes. These genes encode components of the cyst wall (CWP2, 153X higher *in vivo*), enzymes in the UDP-N-acetylgalactosamine biosynthetic pathway (GL50803_14259, 146X higher *in vivo*), and high cysteine membrane proteins (HCMPs, 10X-20X higher *in vivo*). Eight genes with more than 10-fold higher expression in the *in vivo* foci have unknown or uncharacterized functions (Figure 3A and Supplemental Table 4).

Encystation-specific gene expression in *in vivo* foci becomes even more apparent when we consider the functional annotations of genes with more than 2-fold higher expression in *in vivo* foci relative to *in vitro* culture. Thirty-seven differentially expressed genes are associated with encystation, 17 with oxidative stress responses, and 15 with membrane transporter functions (Figure 3B and Supplemental Table 4). This is in contrast to genes highly expressed *in vitro* culture, which include cytoskeletal genes and other genes associated with housekeeping functions such as translation, RNA processing, and transcription (Figure 3C and Supplemental Table 5). Different types of genes with membrane trafficking functions are increased in the two transcriptome datasets. About equal numbers of unique variant specific surface proteins (or VSPs, Nash, 2002) are differentially expressed amongst the *in vitro* (7 VSPs) and *in vivo* foci (8 VSPs) transcriptomes.

Lastly, genes with unknown or uncharacterized function are the most abundant category in both the *in vivo* foci transcriptome (75/187) and the *in vitro* transcriptome (82/166) datasets.

### *In vivo* foci display increased expression of genes associated with the UDP-N-acetylgalactosamine, pentose phosphate, and glycolytic and pyruvate pathways

**Figure 4:**
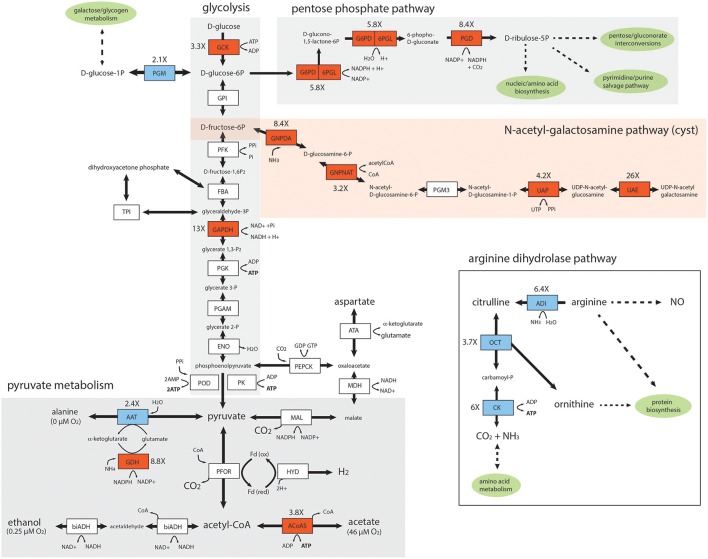
Key enzymes in the glycolytic, pentose phosphate, pyruvate, and UDP-N-acetyl-galactosamine pathways are upregulated in the *in vivo Giardia* foci. Diagrammatic representation of differentially expressed enzymes associated with *in vivo Giardia* energy and biosynthetic pathways. Red shading denotes increased expression of *in vivo* relative to *in vitro* transcripts, and blue shading denotes decreased expression. White shading indicates no significant differential expression between *in vivo* foci and *in vitro* (TYDK) transcriptomes. Oxygen concentrations associated with different branches of the pyruvate metabolic pathway are noted (Lindmark, 1980). The enzyme abbreviations, names, GiardiaDB (GL50803) ORFIDs, and Enzyme Commission numbers are: *Glycolysis*: ACYP, acylphosphatase (7871), EC 3.6.1.7; ENO, enolase (11118), EC 4.2.1.11; FBA, fructose-bisphosphate aldolase (11043), EC 4.1.2.13; GAPDH, glyceraldehyde-3-phosphate dehydrogenase (17043), EC 1.2.1.12; GPI, glucose-6-phosphate isomerase (9115), EC 5.3.1.9; GCK, glucokinase (8826), EC 2.7.1.2; PFK, phosphofructokinase (14993), EC 2.7.1.90; PGAM, phosphoglycerate mutase (8822), EC 5.4.2.1; PGK, phosphoglycerate kinase (90872), EC 2.7.2.3; PGM, phosphoglucomutase (17254), EC 5.4.2.2; PK, pyruvate kinase (17143,3206),EC 2.7.1.40; POD, pyruvate:orthophosphate dikinase (9909), EC 2.7.9.1; TPI, triose phosphate isomerase (93938), EC 5.3.1.1. *Pyruvate metabolism*: ATA, aspartate transaminase (91056), EC 2.6.1.1; FeADH, Fe-alcohol dehydrogenase (3861, 3593) EC 1.1.1.1; ACoAS, acetyl-CoA synthetase (13608); AAT, alanine aminotransferase (12150, 16353), EC 2.6.1.2; biADH (bifunctional alcohol/aldehyde dehydrogenase E (93358), EC 1.1.1.1; GDH, NADP-specific glutamate dehydrogenase (21942) EC 1.4.1.3, 1.4.1.4; Fd, ferredoxin (9662, 27266,10329); HYD, hydrogenase (6304), EC 1.12.7.2; MAL, malic enzyme (14285), EC 1.1.1.38; MDH, malate dehydrogenase (3331), EC 1.1.1.37; PEPCK, phosphoenolpyruvate carboxykinase (10623), EC 4.1.1.32; PFOR, pyruvate:ferredoxin oxidoreductase (17063, 114609); *Pentose Phosphate Pathway (PPP)*: G6PD-6PGL, glucose-6-phosphate-1-dehydrogenase (8682), EC 1.1.1.49; PGD, phosphogluconate dehydrogenase (14759), EC 1.1.1.44; *UDP-N-acetylgalactosamine (GalNac) biosynthetic pathway*: GNPDA, glucosamine-6-phosphate deaminase (8245), EC 3.5.99.6; GNPNAT, glucosamine 6-phosphate *N*-acetyltransferase (14651), EC 2.3.1.4; PGM3, phosphoacetylglucosamine mutase (16069), EC 5.4.2.10; UAE, UDP-*N*-acetylglucosamine 4-epimerase (7982), EC 5.1.3.2; UAP, UDP-*N*-acetylglucosamine diphosphorylase (16217), EC 2.7.7.23. *Arginine Dihydrolase Pathway*: ADI, arginine deiminase (112103), EC 3.5.3.6; ARG-S, arginyl-tRNA synthetase (10521), EC 6.1.1.19; CK, carbamate kinase (16453), EC 2.7.2.2; NOS, nitric oxide synthase (91252), EC 1.14.13.39; OCD, ornithine cyclodeaminase (2452), EC 4.3.1.12; OCT, ornithine carbamoyltransferase (10311), EC 2.1.3.3; ODC, ornithine decarboxylase (94582), EC4.1.1.17; PRO-S, prolyl-tRNAsynthetase (15983), EC 6.1.1.15.

To evaluate the degree to which the differential gene expression we observed is associated with specific metabolic pathways in *Giardia*, we mapped metabolic genes to four catabolic and biosynthetic pathways (Figure 4) using manual curation, as well as GO term and metabolic pathway enrichment tools available from GiardiaDB (Aurrecoechea et al., 2009). Specifically, we mapped the transcriptional upregulation of genes associated with *in vivo* foci to glycolysis and pyruvate metabolism, the pentose phosphate pathway (PPP), the UDP-N-acetylgalactosamine pathway, and the arginine dihydrolase pathway (Figure 4). First, we noted that in *in vivo* foci, the three key enzymes of the arginine dihydrolase pathway (OCT, ADI, CK) showed 3.7–6.4X less gene expression in *in vivo* foci than in trophozoites grown in *in vitro* culture.

The carbohydrate component of the cyst is comprised of a D-GalNAc Beta (1,3)-D-GalNAc homopolymer synthesized from fructose 6-phosphate via the N-acetylgalactosamine (GalNAc) biosynthetic pathway (Figure 4). As has been seen previously during the transcriptional activation of GalNAc pathway enzymes during *in vitro* encystation (Macechko et al., 1992; Jarroll et al., 2001), we found that almost all enzymes in this pathway were expressed between 3.2 and 26X higher in the *in vivo* foci as compared to in the *in vitro* transcriptomes. In fact, the second enzyme in this pathway, glucosamine 6-phosphate *N*-acetyltransferase (GL50803_14651), is one of the top 25 highly expressed genes in *in vivo* foci (Figure 3B and Supplemental Table 2).

Key enzymes involved in the generation of ATP via glycolysis (GCK, 3.3X, and GAPDH, 13X) and pyruvate metabolism (AcoAS, 3.8X) were also more highly transcribed in the foci. PGM expression was several fold (2.1X) lower in the foci transcriptome relative to *in vitro* transcriptome. Pyruvate metabolism in *Giardia* is sensitive to oxygen concentration; at lower oxygen concentrations, pyruvate is fermented to alanine and ethanol, whereas at higher oxygen concentrations pyruvate is converted to acetate with the concomitant production of ATP (Lindmark, 1980). In foci, we observed increased expression of AcoAS, a key enzyme in the higher oxygen pathway resulting in acetate production. We also noted that AAT, involved in alanine production, was less expressed in foci than *in vitro* culture (2.4X). Lastly, two key enzymes of the PPP (G6PD, 5.8X, and PGD, 8.4X) are more highly expressed in the *in vivo* foci transcriptomes (Figure 4).

### Partitioning analysis identifies highly expressed *In vivo* foci genes associated with encystation

**Figure 5:**
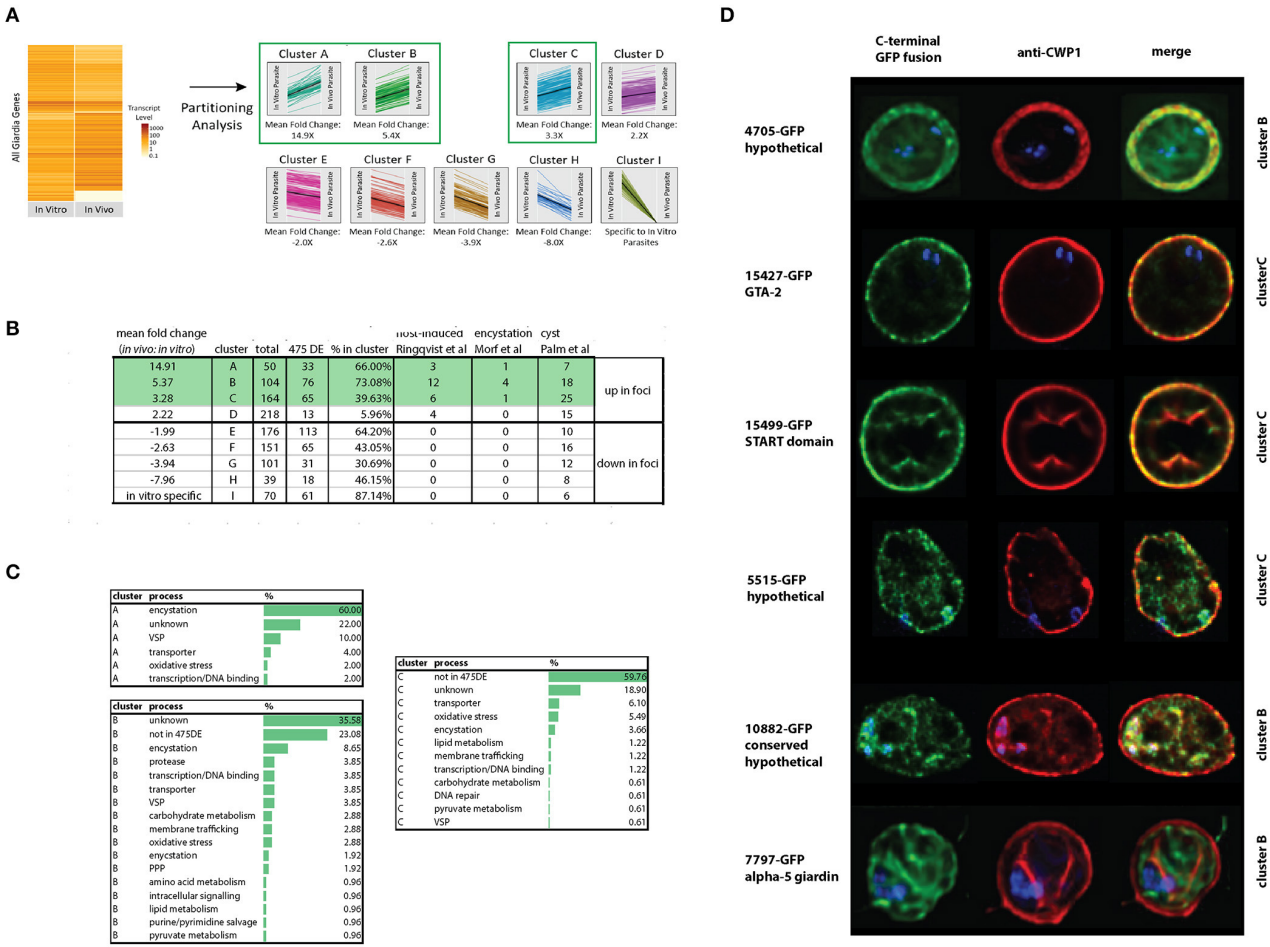
Identification and confirmation of novel proteins expressed highly *in vivo* that localize to cysts. Differentially expressed genes from the *in vivo* foci and *in vitro* culture datasets were partitioned into nine gene clusters (A-I) based on JS distance (Materials and Methods). Many of the 475 differentially expressed genes classified using both Cuffdiff and Sleuth (Materials and Methods) are highly represented in the clusters from this study **(A)**. Other host-induced or encystation-specific transcriptome studies (Palm et al., 2005; Roxstrom-Lindquist et al., 2005; Morf et al., 2010) have fewer genes represented in the clusters than those identified by partitioning analysis in the foci **(B)**. Many genes in clusters A, B, and C are associated with encystation or oxidative stress **(C)**. In **(D)**, C-terminal GFP fusions of genes from cluster B and C also are associated with the cyst wall or the interior of the cyst as visualized using colocalization immunofluorescent images with the cyst wall protein 1 (CWP-1) antibody.

Partitioning analysis sorted nine gene clusters of similar expression levels, and each cluster ranged from 39 to 218 differentially expressed genes (Figure 5A). On average, about 50% of the genes identified using the differential expression analyses (Materials and Methods) were shared with genes in clusters identified using the partitioning analysis (Figure 5B). The gene clusters that were the most highly transcribed in the foci (cluster A, 15X, cluster B, 5X, and cluster C, 3X, respectively) were enriched for encystation, oxidative stress response, and membrane transporter genes (Figure 5C). Cluster A included the most encystation-associated genes (Figure 5B and Supplemental Tables 7,8). These three clusters also shared genes in common with several recent studies of *in vitro* encystation genes (Morf et al., 2010), putative cyst-specific genes (Palm et al., 2005), and putative host-induced parasite genes (Roxstrom-Lindquist et al., 2005; Figure 5B).

### Confirmation of six new cyst-associated proteins expressed at higher levels in the *In vivo* foci

To evaluate whether some of the genes of unknown function identified using either differential expression or partitioning analyses were associated with encystation, we tagged 15 genes using C-terminal GFP tags and created GFP-fusion strains (Dawson and House, 2010). Based on fluorescence microscopy of trophozoites of these GFP fusion strains, 13 of the 15 strains had interphase localization in trophozoites (Supplemental Figure 1). Six had localization in cysts (Figure 5D), with three localizing to the cyst wall: GTA-2 (15427), a START domain protein (15499), and one hypothetical protein (4705). Three GFP fusions localized to the interior region of the mature cyst including alpha-5-giardin (7797), a conserved hypothetical protein (10882), and one hypothetical protein (5515). The 4705-GFP and 7797-GFP strains lacked any localization in the trophozoite stage. Fifteen other genes more highly expressed *in vitro* culture were also tagged, and 14 had localizations in trophozoites (Supplemental Figure 2).

## Discussion

Defining the dynamics of *in vivo* encystation is essential toward understanding *Giardia's* metabolic interactions with the host and commensal microbiota (Morf et al., 2010; Faso et al., 2013; Sulemana et al., 2014). By directly imaging *Giardia* infections using *in vivo* and *ex vivo* bioluminescent imaging (BLI) of *Giardia* with integrated firefly luciferase bioreporters of metabolism (*P*_*GDH*_-*Fluc* strain) or encystation (*P*_*CWP1*_-*Fluc* and *P*_*CWP2*_-*Fluc* strains), we recently showed that parasites colonize both the proximal and distal small intestine in high-density foci early in infection in both mice and gerbils. Encystation is also initiated early during infection in these foci, and persists throughout the entire course of infection (Barash et al., 2017b). The encystation-specific CWP1 and CWP2 promoter fusions to luciferase were highly expressed in the high density *in vivo* foci, and trophozoites in the foci possessed numerous encystation specific vesicles (ESVs) (Barash et al., 2017b). This work challenged the paradigm that encystation is triggered late in infection via spatially segregated cues in the gastrointestinal tract (Barash et al., 2017b), and rather indicated a density-dependent contribution to the initiation of encystation (Barash et al., 2017b).

### Genes associated with encystation are significantly upregulated in the *In vivo Giardia* foci

Using two different analytic methods (Figure 2), we determined that the transcriptome profiles of *Giardia* from *in vivo* foci and from log phase axenic cultures were significantly different. Despite our deriving the samples from the gastrointestinal tracts of four different animals either 3 or 7 days post *Giardia* infection, the transcriptomic profiles of the *in vivo* foci were strikingly similar (Figure 2A). Relative to *in vitro* culture, many of the most highly expressed genes in the *in vivo* foci have functions associated with encystation and with oxidative stress responses. In contrast, the top *in vitro* expressed genes are associated with translation, cell division, and arginine metabolism (Figures 2D,E and Edwards et al., 1992; Stadelmann et al., 2012).

**Figure 6:**
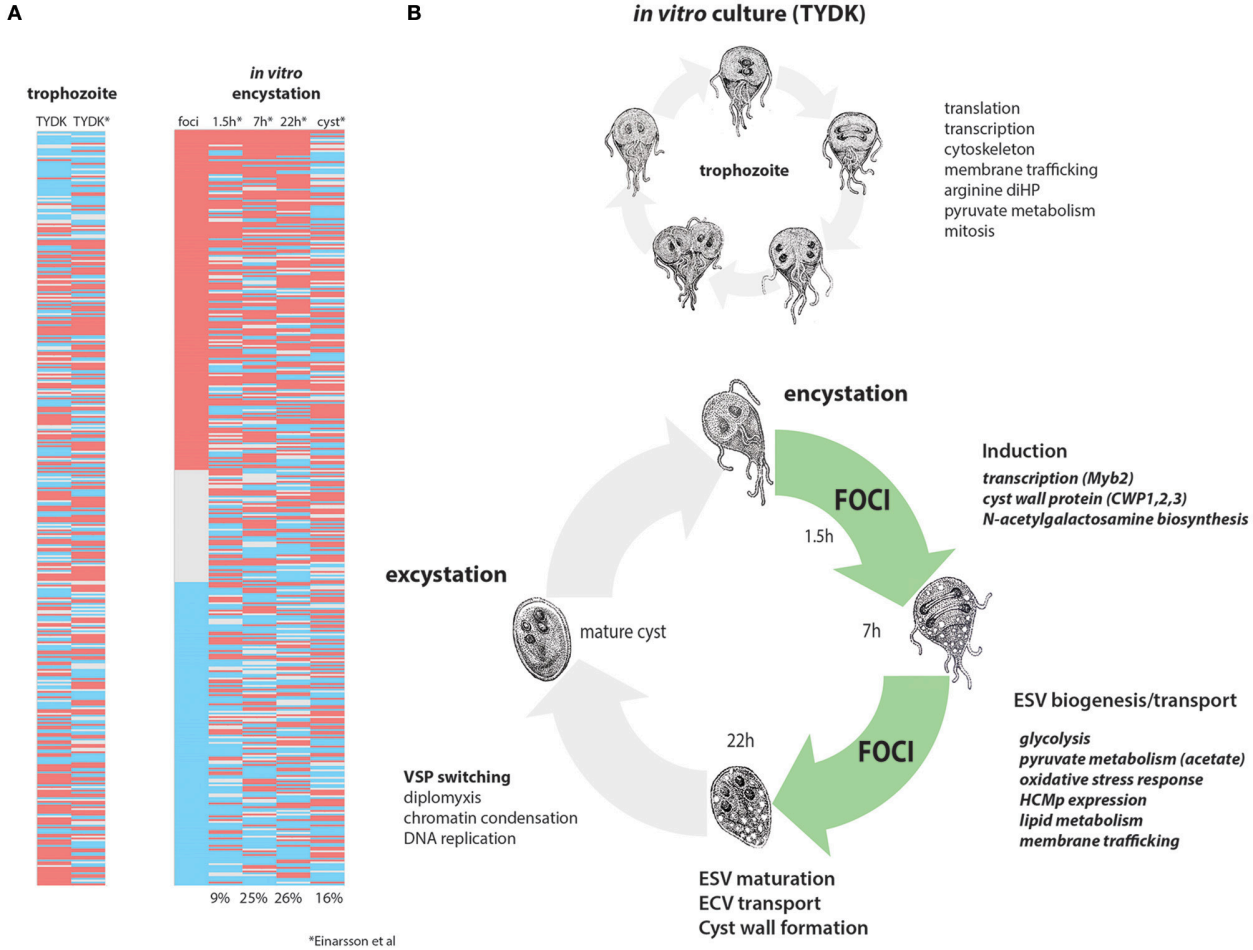
Differentially expressed genes in *in vivo* foci are similar to mid-late encystation time points in *in vitro* encystation transcriptomes. *In vitro* and *in vivo* differentially expressed genes from this study are compared to the *in vitro* encystation transcriptome of Einarsson et al. (2016). **(A)** Compares the 475 differentially expressed genes in this study (red, upregulated in foci; blue, downregulated in foci) to the *in vitro* culture transcriptome (TYDK) and the transcriptomes of four time points of an *in vitro* encystation transcriptome study. Percentages indicate the number of similarly differentially expressed genes in the *in vivo* foci relative to that *in vitro* encystation time point. **(B)** Summarizes processes upregulated in *in vitro* culture, which are primarily trophozoite growth and division (above) as contrasted with cellular processes upregulated in the *in vivo* foci (green) that are more similar to those genes upregulated during in mid-to-late *in vitro* encystation.

Landmark *in vitro* studies established that the initiation of encystation is transcriptionally controlled (Upcroft et al., 1996; Barat and Bloland, 1997; Land and Johnson, 1999) by a Myb transcription factor within 90 min of switching trophozoite cultures to encystation medium (Sun et al., 2002). GalNAc enzyme transcription has been shown to peak at about 22 h in encystation medium (Einarsson et al., 2016). Trophozoites in each *in vivo* focus are likely committed to encystation (Barash et al., 2017b), as the transcriptome of each *in vivo* focus is defined by the high expression of genes associated with later stages of encystation and cyst wall biosynthesis. In total, we have confirmed the expression of 37 known encystation genes and identified six new cyst–associated proteins in the high-density foci (Figure 6). Because we have identified many highly expressed encystation-associated transcripts in key metabolic, biosynthetic, protease, and membrane trafficking pathways, we suggest that the majority of cells in the high-density foci in the proximal intestine are in mid- to late stage encystation (Figure 6 and Supplemental Table 9). The apparent developmental synchrony of cells in the foci is supported by the strong encystation transcriptional profiles in each sample regardless of the day of infection (Figure 6) or in each animal sampled (Figure 2A) and may be the result of density related cues such as nutrient or lipid deprivation that trigger encystation.

In support of the idea that *Giardia* in the foci are encysting, we noted significantly higher expression of the master transcriptional regulator Myb2, all enzymes of the GalNAc biosynthetic pathway (Figure 4), two known cyst wall proteins (CWP2 and CWP3), 17 HCMPs, and two EGF family proteins (Chen et al., 1996; Figures 2, 3, and Supplemental Table 4) in the *in vivo* foci. CWP1 (Ebneter et al., 2016) was identified as differentially expressed by only one of the two methods of analysis we used, and was thus not included in the 475 differentially expressed genes (Materials and Methods). Through partitioning analysis and analysis of differential gene expression in the *in vivo* foci, we also identified six new cyst-specific proteins (Figure 5D and Supplemental Tables 7,8). Each localizes to the cyst wall or the cyst interior after 24 h of incubation in encystation medium.

Variant-specific surface proteins (VSPs) contribute to *Giardia's* evasion from the host immune response (Prucca and Lujan, 2009), and only one VSP is typically expressed per cell (Li et al., 2013). VSP expression occurs early during *in vitro* encystation and VSP switching occurs during later stages (Prucca et al., 2008; Einarsson et al., 2016). Fourteen VSPs were differentially expressed in our analysis, and two VSPs were among the most highly expressed proteins in *in vivo* foci relative to *in vitro* culture (Figure 2).

*Giardia* directly perturbs lipid metabolism within the host by scavenging the gut lumen for lipids and by excreting novel end products of lipid metabolism (Mendez et al., 2015). *Giardia* also contributes to shifts in the local gut microbiota (Halliez and Buret, 2013) by both excreting novel lipids and influencing the bioavailability of bile acids (Gillin et al., 1986; Das et al., 1997, 2002; Yichoy et al., 2011). Lipids also play key roles in the regulation of encystation (Mendez et al., 2015). Glucosylceramide transferase-1 (gGlcT1), an enzyme involved in sphingolipid biosynthesis, is highly expressed in *in vivo* foci and has also been shown to play a key role in ESV biogenesis and cyst viability (Sonda et al., 2008). Also highly expressed in foci is the acid sphingomyelinase-like phosphodiesterase 3b precursor (gSmase 3B) which is also transcriptionally upregulated during encystation and may scavenge ceramide in the small intestine (Sonda et al., 2008). We also noted the increased *in vivo* expression of seven genes associated with membrane trafficking that could be involved in ESV biogenesis or transport (Figure 3 and Supplemental Table 4).

In *Giardia*, cathepsin cysteine proteases are linked not only to encystation (Touz et al., 2002) but also to evasion of innate and adaptive immune responses. In co-culture with rat IEC cells, one (3099) of the nine cathepsin B proteases genes has significantly increased expression *Giardia* trophozoites (Ma'ayeh and Brook-Carter, 2012). Five cysteine proteases (three cathepsin B homologs: 10217, 17516, 16779 and two cathepsin L homologs: 11209, 137680) have roughly five and 11.5-fold higher expression in the foci (Supplemental Tables 2,4). Cathepsin B (16779, CP3) and cathepsin L (137680) are known to be upregulated in encysting trophozoites (DuBois et al., 2008).

### Energy metabolism of trophozoites encysting in the *In vivo* foci

As an anaerobic fermentative microbe, *Giardia* derives energy from glycolysis, pyruvate fermentation and the arginine dihydrolase pathway (Troeger et al., 2007 and see Figure 4). Though trophozoites cannot tolerate high oxygen concentrations, they actively consume oxygen (Paget et al., 1993a,b) and may take advantage of slightly elevated oxygen levels for energetic benefit (Paget et al., 1990; Edwards et al., 1992; Adam, 2001). As oxygen levels increase above 0.25 μM, the ATP producing arm of pyruvate metabolism is activated and acetate and ATP become primary end products, with the highest NAD(P)+ or ATP yields occurring at the higher end of tolerated oxygen concentrations (Schofield et al., 1990, 1991, 1992; Edwards et al., 1992). We noted higher expression of genes associated with energy metabolism via glycolysis (GCK, 3.3X and GAPDH, 13X) and pyruvate fermentation via pathways associated with higher oxygen tensions (Figure 4). These expression patterns are indicative of redox stress and encystation-specific metabolism associated with the *in vivo* foci. At higher oxygen concentrations, it is hypothesized that antioxidant enzymes consume NAD(P)H to neutralize dO_2_ and ROS/RNS, leading to regeneration of NAD(P) for glycolysis, allowing pyruvate to be used for ATP production, instead of NAD(P)H regeneration (Upcroft et al., 1996). In the *in vivo* foci, acetate could be the primary end product of *Giardia* fermentation due to the relatively increased expression of ACoAS (3.8X) and the decreased expression of AAT (2.4X) we observed (Figure 4). Thus, parasites in the high-density foci may take advantage of a more oxidized environment to switch to more energetically favorable pyruvate fermentation. We predict the increased *in vivo* expression of GDH is associated with parasite ammonia assimilation and the cycling of NAD(P) to maintain intracellular redox balance (Yee et al., 2000).

Encysting trophozoites slow their catabolism of glucose for energy and switch to using glucose to synthesize N-acetylgalactosamine for cyst wall synthesis (Jarroll et al., 2001 and Figure 4). GalNAc is synthesized only during encystation via a transcriptionally induced pathway of five enzymes that produces UDP-GalNac from fructose-6-phosphate that is diverted from ATP production in glycolysis to cyst wall polysaccharide biogenesis. This diversion could result in a net loss of ATP synthesis during encystation.

While little is known about the regulation of glycolysis and energy production during encystation, it is thought that additional ATP is generated from the arginine dihydrolase pathway (ADiHP) (Figure 4). This pathway yields ATP via the conversion of arginine to ammonia and citrulline, with the substrate-level phosphorylation of citrulline yielding ornithine and carbamoylphosphate, and resulting in NH_3_, CO_2_ as end products (Schofield et al., 1992). Ornithine carbamoyltransferase (OCT) is one of the most highly expressed enzymes in culture and in the *in vivo* foci (Figure 2), yet we observed less expression of key enzymes in the ADiHP relative to *in vitro* culture (Figure 4). This is counter to the prevailing notion that encysting trophozoites increase arginine catabolism through the ADiHP to offset energy lost from slowing glycolysis (Stadelmann et al., 2012).

Lastly, trophozoites also lack purine and pyrimidine synthesis, relying solely on salvage pathways to obtain these metabolites from the host and commensal microbiota (Jarroll et al., 1981; Wang and Aldritt, 1983; Aldritt et al., 1985). We also discovered that several key enzymes of the PPP (G6PD 5.8X and PGD 8.4X) have increased expression in the *in vivo* foci transcriptome (Figure 4). A key product of the PPP is NADPH, which plays an integral role in reductive biosynthesis as well as in the defense against oxidative stress and the maintenance of cellular redox. In *Giardia* and in *Trichomonas*, the G6PD and 6PGL enzymes are fused and are directly involved in the oxidative branch of the PPP in generating reduced NADPH (Figure 4 and Stover et al., 2011). This suggests that in the foci, there is increased cycling of reducing equivalents with CO_2_ production and the increased production of D-ribose-5P as a key substrate for nucleotide and amino acid biosynthesis and pyrimidine/purine salvage pathways.

### Oxidative stress responses are increased in the *In vivo* foci

Transcriptomic analyses of the high-density foci suggest that they are susceptible to and are responding to localized oxidative and nitrosative stresses (Brown et al., 1996; Di Matteo et al., 2008). *Giardia* attaches to the villi of the small intestine, where oxygen concentrations are up to 60 μM (Adam, 2001). Anti-oxidant defense is thought to be mediated by the thioredoxin-thioredoxin reductase system, as *Giardia* lacks superoxide dismutase, catalase, and glutathione-glutathione reductase. The thioredoxin-thioredoxin reductase system includes an FAD containing NADPH-dependent disulfide reductase, a thioredoxin, a thioredoxin reductase (TrxR) and a thioredoxin-peroxidase (Faghiri and Widmer, 2011). In oxidative environments, *Giardia* expresses NADH oxidase and flavodiiron protein that metabolize oxygen to form water (Brown et al., 1996; Di Matteo et al., 2008). Nitrosative stresses can have differing metabolic effects, however; while high micromolar concentrations of nitric oxide are toxic (Fernandes and Assreuy, 1997), lower levels inhibit cell proliferation, encystation, and excystation (Eckmann et al., 2000). Resolution of nitric oxide stress by *Giardia* is believed to be mediated by activity of its flavohemoglobin protein (Rafferty et al., 2010), which catalyzes the oxidation of nitric oxide to nitrate using oxygen as a co-substrate (Gardner et al., 1998; Hausladen et al., 1998; Gardner, 2005; Ilari and Boffi, 2008).

In particular, we observed the increased expression of genes associated with oxidative or nitrosative stress and detoxification, including NADPH oxidoreductase (17150, 15004), nitroreductase (FMN) family protein (8377), and flavohemoglobin (15009) at levels that were 2.5–8.5-fold higher than standard *in vitro* culture (Mastronicola et al., 2010). Indeed, many of the top 25 expressed genes in the *in vivo* foci (e.g., NADPH oxidoreductase) are associated with oxidative or nitrosative stress responses (Ma'ayeh et al., 2015), which may explain how *Giardia* can colonize the proximal part of the small intestine, a fairly aerobic portion of the intestinal tract (Mastronicola et al., 2010). Due to these transcriptomic patterns, we predict that *Giardia* trophozoites in high-density *in vivo* foci are more likely to be subject to host-induced oxidative stressors than *Giardia* growing at lower density.

Thioredoxin associated proteins also aid in the detoxification of oxidative stress, and five thioredoxin domain proteins (9827, 6289, 2388, 8064, 9335) were also highly expressed in the *in vivo* foci (Figures 2, 3). One thioredoxin reductase (TrxR, 9827) has disulfide reductase activity and converts oxidized thioredoxin to its reduced form. Reduced thioredoxin is, in turn, used to prevent protein misfolding that can occur under oxidative stress conditions (Land and Johnson, 1999). *Giardia's* TrxR also has NADPH oxidase activity, and during conversion of thioredoxin to its reduced form, NADP is generated from NADPH (Gillin et al., 1989).

### Density dependence of encystation and the consequences of encystation on host pathology

The transcriptional profiles of the sampled foci resemble the transcriptional profiles seen for *in vitro* encysting trophozoites, suggesting the encysting trophozoites in the foci have received the inducing encystation stimulus within the last 12 h (Sulemana et al., 2014). We hypothesize that once parasites reach threshold high densities in discrete regions of the gut, they could trigger localized host immune responses and redox shifts, resulting in the observed increase in oxidative stress and encystation responses that define transcription in the foci. Current hypotheses about how *Giardia* induces host symptoms have focused primarily on specific damage induced by parasite attachment to the host gut epithelium or on the production of anti-microbial metabolites by the host. The physiology of parasites within the high-density foci in the host gut clearly differs from that of cells in laboratory culture or in co-culture with cell lines. The observed encystation- and oxidative stress-specific responses in gene expression within parasite foci likely reflect the physiological changes associated with high-density growth in localized regions of the gut. *Giardia* infection results in a dysbiosis throughout the gastrointestinal tract in mice, characterized by a shift in the diversity of commensal microbiota toward more aerobic Proteobacteria species (Barash et al., 2017a). As we find that encystation-specific metabolism occurs early and consistently during *Giardia* infection in the host, it is possible that the crowding, nutrient deprivation and accumulation of waste products that likely trigger encystation would also affect the nutrition of the host and associated commensal microbiome. Further investigation of *Giardia*-host-microbiome interactions should thus emphasize the study of such interactions *in situ* in order to vet laboratory culture and co-culture studies.

## Ethics statement

This study was carried out in accordance with the recommendations of the IACUC Committee at the University of California, Davis. The protocol was approved by the UC Davis IACUC Committee.

## Author contributions

JP processed samples, analyzed transcriptome data, imaged GFP strains, and created figures and tables, and wrote the manuscript. CN designed animal experiments, collected and analyzed BLI images, and imaged GFP strains. ES analyzed transcriptome data and created figures and tables. KN created and imaged GFP strains. KH created and imaged GFP strains, and edited the manuscript. HS analyzed transcriptome data and developed metabolic models. SD designed experiments, annotated genes, created figures, and wrote and edited manuscript.

### Conflict of interest statement

The authors declare that the research was conducted in the absence of any commercial or financial relationships that could be construed as a potential conflict of interest.

## Supplementary material

Supplemental Figure 1 Subcellular localization of C-terminal GFP fusion proteins of selected genes with increased expression in the *in vivo* foci.

Supplemental Figure 2 Subcellular localization of C-terminal GFP fusion proteins of selected genes with increased expression in *in vitro* axenic culture.

Supplemental Table 1 Quantification of whole body and *ex vivo* bioluminescent imagine at day 3 and day 7 p.i. for transcriptomic sampling of foci.

Supplemental Table 2 FPKM of all genes in the *in vivo* and *in vitro* transcriptomes.

Supplemental Table 3 Overall heatmap of all FPKM.

Supplemental Table 4 Heat map table unregulated.

Supplemental Table 5 Heat map table - down regulated.

Supplemental Table 6 Heat map table - *in vitro* only.

Supplemental Table 7 Cuffdiff partitioning analysis into nine clusters.

Supplemental Table 8 Annotated nine clusters.

Supplemental Table 9 Comparison of *in vivo* foci and *in vitro* encystation transcriptomes.
