# Supplementary figures and images for: Transcriptomic Profiling of High-Density Giardia Foci Encysting in the Murine Proximal Intestine
Source: Front Cell Infect Microbiol. 2017 May 31;7:227. doi: 10.3389/fcimb.2017.00227 (PMC5450421; doi:10.3389/fcimb.2017.00227)

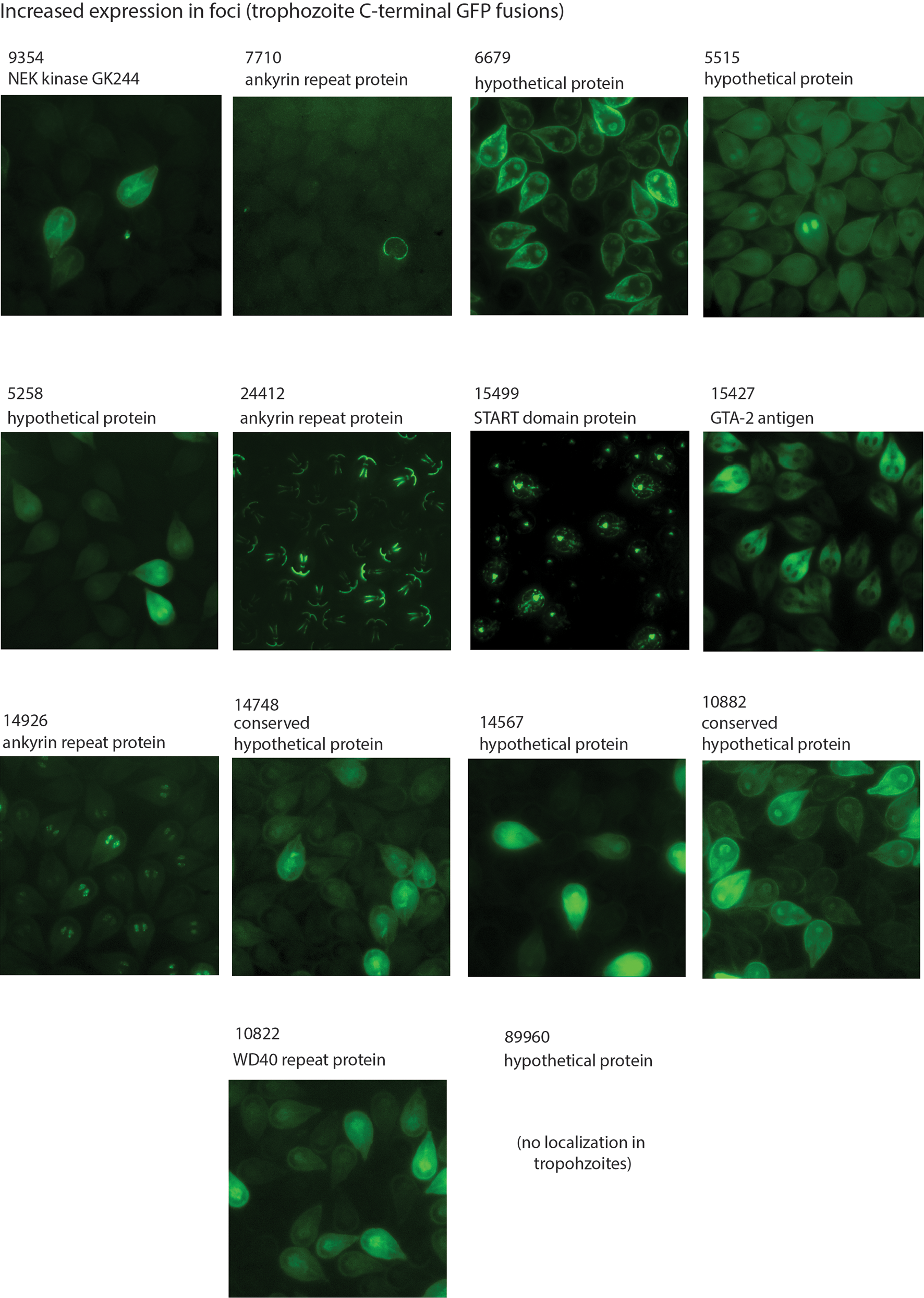

Supplement: Supplemental Figure 1 — Subcellular localization of C-terminal GFP fusion proteins of selected genes with increased expression in the in vivo foci. [file Image1.TIF]

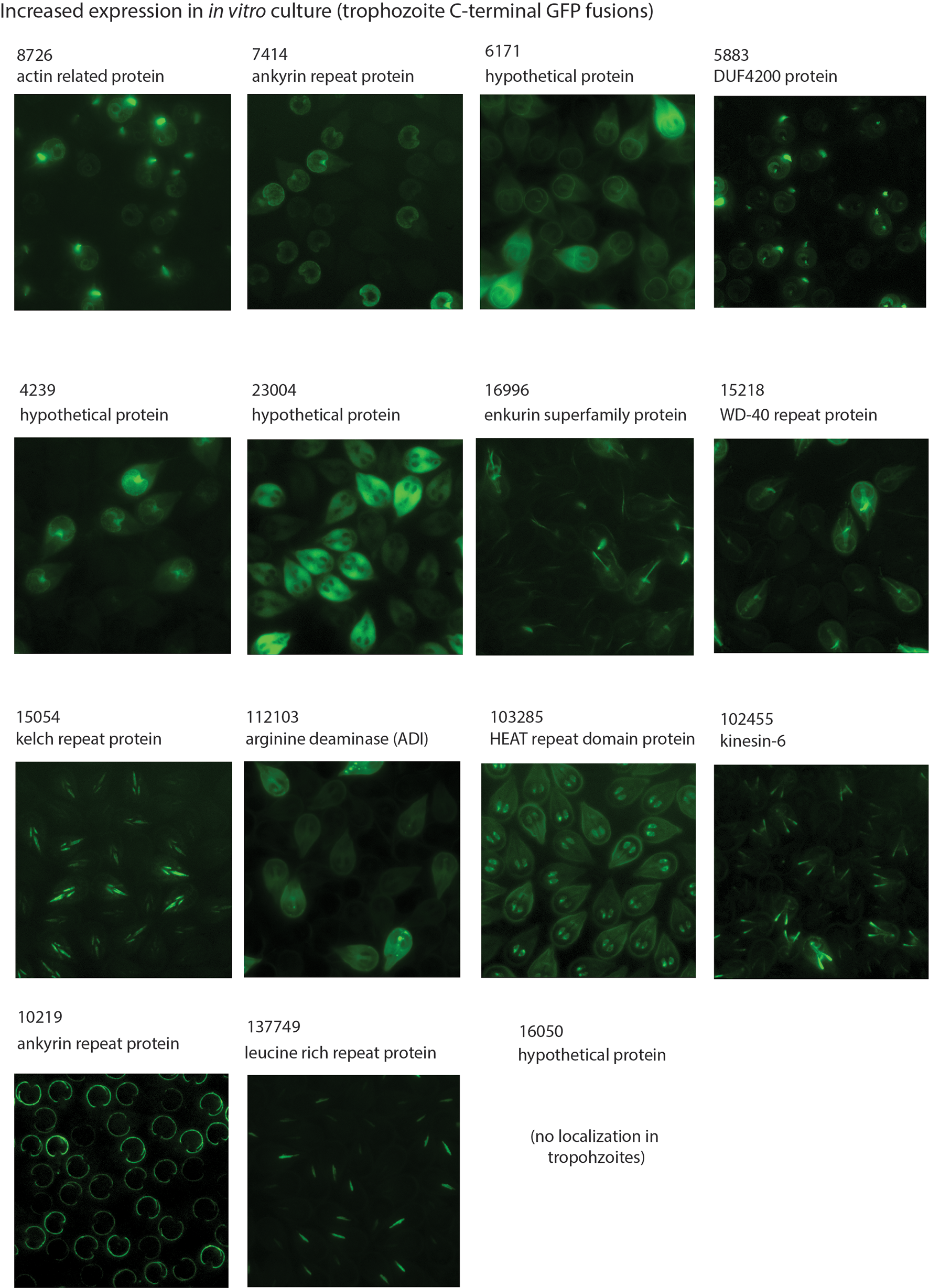

Supplement: Supplemental Figure 2 — Subcellular localization of C-terminal GFP fusion proteins of selected genes with increased expression in in vitro axenic culture. [file Image2.TIF]
